# Parallel Genomic Engineering of Two *Drosophila* Genes Using Orthogonal *attB/attP* Sites

**DOI:** 10.1534/g3.118.200565

**Published:** 2018-08-07

**Authors:** Beatriz Blanco-Redondo, Tobias Langenhan

**Affiliations:** Rudolf Schönheimer Institute of Biochemistry, Division of General Biochemistry, Medical Faculty, Leipzig University, 04103 Leipzig, Germany

**Keywords:** phiC31, transgenesis, *Drosophila melanogaster*, CRISPR, orthogonal

## Abstract

Precise modification of sequences in the *Drosophila melanogaster* genome underlies the powerful capacity to study molecular structure-function relationships in this model species. The emergence of CRISPR/Cas9 tools in combination with recombinase systems such as the bacteriophage serine integrase ΦC31 has rendered *Drosophila* mutagenesis a straightforward enterprise for deleting, inserting and modifying genetic elements to study their functional relevance. However, while combined modifications of non-linked genetic elements can be easily constructed with these tools and classical genetics, the independent manipulation of linked genes through the established ΦC31-mediated transgenesis pipeline has not been feasible due to the limitation to one *attB/attP* site pair. Here we extend the repertoire of ΦC31 transgenesis by introducing a second pair of *attB/attP* targeting and transgenesis vectors that operate in parallel and independently of existing tools. We show that two syntenic orthologous genes, *CG11318* and *CG15556*, located within a 25 kb region can be genomically engineered to harbor *attP^TT^* and *attP^CC^* sites. These landing pads can then independently receive transgenes through ΦC31-assisted integration and facilitate the manipulation and analysis of either gene in the same animal. These results expand the repertoire of site-specific genomic engineering in *Drosophila* while retaining the well established advantages and utility of the ΦC31 transgenesis system.

The amenability of the fruitfly’s genome to targeted manipulation in combination with the vast phenotyping repertoire for this model species has enabled the precise interrogation of gene product functions. Several methodological advances have facilitated the use of directed genomic engineering in the fly. The advent of homologous recombination strategies enabled the exact targeting of genomic sequences in the fly genome, yet the stochastic nature of the occurrence of double-strand breaks (DSB) rendered this method a tedious and time-consuming venture (Gong and Golic 2003). Protocols that have made CRISPR/Cas9 (clustered regularly interspaced short palindromic repeats/CRISPR-associated 9) technology available to *Drosophila* genomic engineering have overcome these limitations, and single or multiple DSBs can since be exactly and efficiently induced at genomic targets (Bassett *et al.* 2013; Gratz *et al.* 2013a; Yu *et al.* 2013; Kondo and Ueda 2013; Ren *et al.* 2013; Sebo *et al.* 2014) . A DSB can either be inaccurately repaired by non-homologous end joining creating a palette of insertion-deletion mutations, the exact sequence of which cannot be controlled by the experimenter. Alternatively, in a gene replacement approach two DSBs release a defined genomic fragment that may harbor an entire gene or part of it (Gratz *et al.* 2013b). The concomittant provision of a DNA template via a homology directed repair (HDR) vector containing homology arms corresponding to the up- and downstream sequences of the released genomic fragment, a selection cassette for the identification of recombinant progeny, and sequences for their further genomic manipulation, offer an elegant means to pre-determine the precise layout of the engineered allele (Gratz *et al.* 2014; Port *et al.* 2014).

The incorporation of an *attP* (phage attachment) recognition site for the *Streptomyces* ΦC31 phage integrase within HDR vectors has become a standard procedure to replace an endogenous locus in *Drosophila* (Huang *et al.* 2009; Gratz *et al.* 2014). Subsequent integration of matching *attB* (bacterial attachment) site-encoding plasmids into *attP*-carrying flies together through germ-line expression of ΦC31 has greatly enhanced the speed, accuracy and reproducibility of fly transgenesis (Groth *et al.* 2004; Bischof *et al.* 2007). Once an *attP* founder fly line is established, ΦC31 transgenesis can be used to generate an unlimited number of allelic variants of the locus by restoring it with modified genomic fragments that contain mutations, in-frame fusions or other modifications of the genetic element of interest. Consequently, ΦC31-mediated transgene insertion enables high-throughput structure-function studies of genes and their products in *Drosophila* at single nucleotide and single amino acid resolution, respectively.

The analysis of genetic pathways, the study of gene homologs and the requirement of independent genomic modifications in the same animal requires multiple concurrent changes to its genome. However, the one-on-one compatibility of the *attB/attP* pair restricts the use of the ΦC31 platform to one locus per fly strain. The targeting of multiple *attP* sites at different genomic positions with non-identical transgenes in the same founder animal is not possible as each *attP*-landing pad is equally receptive to the insertion event. Consequently, if and where each plasmid integrates is stochastic violating the concept of site-specificity of ΦC31 transgenesis, one of its most compelling features. For non-linked genomic targets this problem can be solved by the manipulation of each locus of interest in an individual parental line, and their subsequent genetic combination through crossing. In contrast, linked loci on the same chromosome are not amenable to this option, particularly if their genetic distance is too small and thus the frequency of meiotic recombination to place them on the same chromosome is impracticably low.

Here we present an alternative approach to permit the manipulation and independent genomic engineering of linked loci through established ΦC31 integrase resources. ΦC31 cleaves double-stranded DNA at a central crossover dinucleotide within *attB* and *attP* sites generating a matching two-base pair 5′-*TT* overhang in both (Figure 1A, Table S1). Subsequently, the integrase swaps the half-sites and ligates the reciprocal partners creating hybrid *attL* and *attR* sites (Smith *et al.* 2004a). While the overhangs are essential for the recombination reaction, their sequences are not as long as they remain reverse-complementary to each other (Colloms *et al.* 2014). We have capitalized on this aspect of ΦC31 integration and adopted a matched *attB/attP* pair whose crossover dinucleotide consists of two cytosines (Figure 1B, Table S1; here referred to as *attB^CC^/P^CC^*) instead of the commonly used thymines in standard ΦC31 vectors for *Drosophila* (Figure 1A; here referred to as *attB^TT^/P^TT^*). This allowed us to use a selection of established ΦC31 integrase expressing fly strains with high integration efficiency without changes in the integration protocol. Existing plasmids for CRISPR/Cas9-mediated gene replacement and ΦC31 transgenesis were modified to encode the orthogonal *attB^CC^/P^CC^* pair.

**Figure 1:**
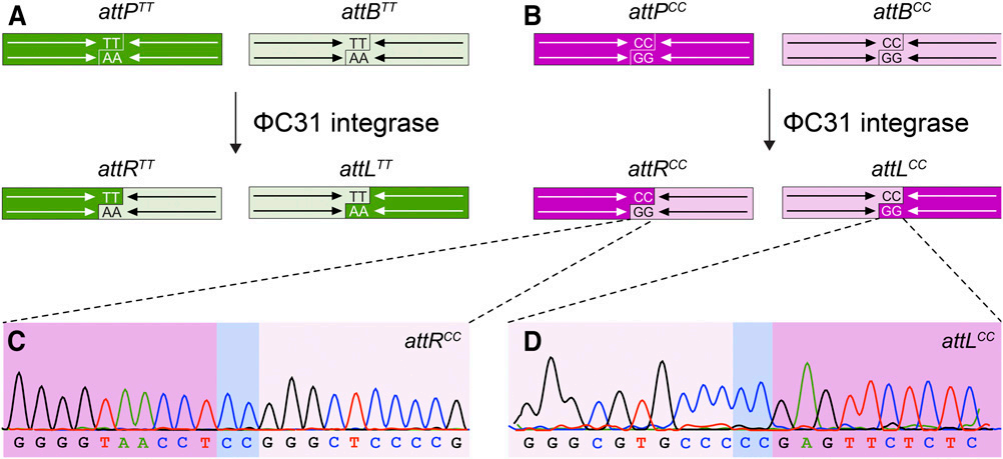
Orthogonal *attP* and *attB* site design. (A) Canonical attP/attB vectors contain a central *TT* dinucleotide at which the ΦC31 integrase-mediated crossover between the two partner sequences occurs. The recombination event leads to the generation of hybrid *attR* and *attL* sites as indicated. (B) The orthogonal *att* site pair contains a *CC* instead of the *TT* sequence as cross-over nucleotide in both *attP* and *attB* vectors. (C,D) Sanger sequencing of genomic DNA of recombinant fly strains after insertion of *attB^CC+^* transgenes into an *attP^CC+^* landing site confirms that ΦC31 catalyzes the recombination between these non-canonical elements leading to the generation of (C) *attR^CC^* and (D) *attL^CC^* sites. Note the *CC* cross-over dinucleotide (boxed in blue) present in both hybrid sites. Chromatograms display the forward strand nucleotide sequence (upper strand in B), which was confirmed by corresponding reverse strand sequencing (lower strand in B; not shown).

This approach permitted the targeting and subsequent genomic engineering of two homologs of the adhesion GPCR (aGPCR) family (Hamann *et al.* 2015), *CG11318* and *CG15556*, which are closely linked on a genomic fragment and separated through intervening genes on chromosome *III*. We show that our approach can be used to sequentially but also simultaneously integrate transgenes in a chromosome endowed with orthogonal *attP* sites using ΦC31 while maintaining efficiency, specificity and directionality of the targeting procedure. We demonstrate the utility of this approach by obtaining the co-transcriptional gene activity pattern of the *CG11318/CG15556* gene pair in *Drosophila*.

## Materials and Methods

### Molecular reagents

All plasmids engineered herein were modified using restriction enzymes from New England Biolabs. PCRs were conducted using AccuStar DNA Polymerase (Eurogentec), primers and custom DNA fragments were synthesized by MWG Eurofins or Life Technologies. All intermediate and final constructs were DNA-sequenced to ensure no errors were introduced during the cloning procedures. The template genomic DNA used for PCR amplification throughout the study was from our stock of the *w^1118^* strain (Flybase ID: FBal0018186).

#### pHD-mW-attP^CC^-FRT (Addgene ID: 115158):

The HDR vector contains a combination of principal elements of the *pHD-DsRed-attP* and *pGX-attP* vectors previously published by (Gratz *et al.* 2014) and (Huang *et al.* 2009). It harbors two multiple cloning sites on both sides of the replacement/mini-white marker element that are flanked by type IIS restriction sites, *Aar*I (5′ MCS) and *Sap*I (3′ MCS), respectively, to seemlessly insert homology arms for homology directed repair after CRISPR/Cas9-mediated cleavage of genomic sequences (Gratz *et al.* 2014). The *mini-White* marker element is flanked by two FRT sites for its subsequent removal by FLP recombinase expression. In addition, the replacement cassette contains a modified *attP* ΦC31 docking site with the central cross-over nucleotides changed from TT to CC (*attP^CC^*). This way, *pHD-mW-attP^CC^-FRT* with its selection marker, marker removal sites and *attP* integration elements can be used in parallel and thus in combination with *pHD-DsRed-attP*.

The vector was generated as follows. A 175 bp DNA fragment containing the *5′-Nde*I-*attP^CC^-FRT-Bsi*WI-*Bam*HI-*FRT-Spe*I*-3′* elements was custom synthesized (pTL706), and inserted into *pHD-DsRed-attP* at the *Nde*I/*Spe*I restriction sites (pTL716) replacing its *attP^TT^-loxP-DsRed-loxP* cassette. Subsequently, the resulting plasmid was opened with *Bsi*WI and *Bam*HI and a 3.0 kb fragment of *pGE-attB* (Huang *et al.* 2009) containing the *GMR-mini-White* cassette was inserted therein generating the final *pHD-mW-attP^CC^-FRT* (pTL717) replacement vector (Figure S1A).

#### pGE-attB^CC^-FRT-mW (Addgene ID: 115159):

The *attB^CC^* integration vector was constructed by synthesizing a 153 bp DNA fragment containing *5′-Sac*II-*attB^CC^-FRT-Bsi*WI-*3′* elements (pTL705). This fragment was then inserted into the *pGE-attB* plasmid (Huang *et al.* 2009) after a *Sac*II and *Bsi*WI double digest (pTL788; Figure S1B). The central cross-over nucleotides of the *attB* ΦC31 site are changed from *TT* to *CC* (*attB^CC^*).

#### pGE-attB^TT^-loxP-DsRed (Addgene ID: 115160):

A 1.2 kb PCR fragment containing the *loxP-3xP3-DsRed* selection/integration cassette was amplified off *pHD-DsRed-attP* (Gratz *et al.* 2014) using primers tl_814F/tl_815R, the amplicon was cut with *Bsi*WI/*Bgl*II and ligated into the 3.1 kb *Bsi*WI/*Bam*HI fragment of *pGE-attB* (Huang *et al.* 2009) to generate *pGE-attB^TT^-loxP-DsRed* (pTL780; Figure S1C).

#### pU6-gRNAs:

CRISPR/Cas9 cutting sites 5′ and 3′ of the *CG11318* and *CG15556* loci suitable to remove all exons, UTRs and the promoter regions were identified by ‘CRISPR Optimal Target Finder’ (Gratz *et al.* 2014) (Table 1). The genomic sequence of all CRISPR/Cas9 cleavage sites were confirmed by DNA sequencing of PCR fragments encompassing the suggested sites prior to cloning. Target-specific sequences for *CG11318* and *CG15556* gRNAs were synthesized as 5′-phosphorylated oligonucleotides, annealed, and ligated into the *Bbs*I sites of the *pU6-BbsI-chiRNA* vector (Gratz *et al.* 2013a).

**Table 1:** pU6-gRNA plasmid cloning for CG15556 and CG11318 targeting

Gene	Site	chiRNA plasmid	Primers used
*CG15556*	5′ cut	pTL633	tl_657F/tl_658R
	3′ cut	pTL634	tl_659F/tl_660R
*CG11318*	5′ cut	pTL635	tl_661F/tl_662R
	3′ cut	pTL636	tl_663F/tl_664R

#### CG11318 HDR vector:

A 0.9 kb fragment encoding the 5′ homology arm was amplified from genomic DNA using primers tl_681F/682R, cut with *Aar*I and inserted into de-phosphorylated *Aar*I-digested *pHD-DsRed-attP* (pTL645). Subsequently, the 1.2 kb 3′ homology arm was PCR-amplified from genomic DNA using primers tl_683F/684R, cut with *Sap*I, and inserted into de-phosphorylated pTL645 to generate the final *CG11318* targeting vector pTL650 (*attP^TT+^*, *loxP^+^*, *DsRed^+^*).

#### CG15556 HDR vector:

A 0.9 kb 5′ homology arm fragment was amplified from genomic DNA using primers tl_673F/674R, cut with *Aar*I and inserted into de-phosphorylated *Aar*I-digested vector *pHD-mW-attP^CC^-FRT* (pTL722). Then, a 1.4 kb 3′ homology arm fragment was amplified from genomic DNA using primers tl_675F/676R, cut with *Sap*I and inserted into de-phosphorylated *Sap*I-digested vector pTL722 to generate the final *CG15556* targeting vector pTL724 (*attP^CC+^*, *FRT^+^*, *mW^+^*). For primer design see also Figure S1D.

#### CG11318-GAL4 reporter vector:

A 4.6 kb fragment corresponding exactly the the genmic *CG11318* sequence removed through the CRISPR/Cas9 cuts was amplified off genomic DNA with primers tl_768F/tl_769R, which contained *Not*I and *Asc*I restriction sites, respectively. The DNA fragment was double digested with *Not*I and *Asc*I and inserted into *pGE-attB^TT^-DsRed* to generate a wild-type *CG11318* rescue vector (pTL784). In order to insert a GAL4.2 transcription factor cassette at the transcriptional start site of *CG11318*, a 1.6 kb *Age*I/*Nsi*I fragment of pTL784 was subloned into pTL550 (*pMCS5* derivative with *KanR*; MoBiTec; pTL785). This subclone was outward PCR-amplified using primers tl_824F/tl_825R to generate a 4.6 kb amplicon. An 1.6 kb fragment encoding the optimized GAL4 cassette was amplified off *pBPGal4.2*::*p65d* (Pfeiffer *et al.* 2010) using primers tl_822F/tl_823R. Both PCR fragments were appended with primer-encoded *Bgl*II and *Nhe*I sites on either end, respectively, digested with *Bgl*II/*Nhe*I and ligated generating clone pTL787. A 3.2 kb *Age*I/*Nsi*I fragment of this clone was re-transferred into the *CG11318* rescue vector pTL784 to construct the final *CG11318-GAL4* reporter allele plasmid pTL789 (*attB^TT+^*, *loxP^+^*, *DsRed^+^*).

#### CG15556 LexA reporter vector:

A 4.0 kb fragment corresponding exactly to the genomic *CG15556* sequence removed through the CRISPR/Cas9 cuts was amplified off genomic DNA with primers tl_827F/tl_828R, which contained *Not*I and *Asc*I restriction sites, respectively. The DNA fragment was double digested with *Not*I and *Asc*I and inserted into *pGE-attB^CC^* (pTL788) to generate a wild-type *CG15556* rescue vector (pTL790). In order to insert a LexA transcription factor cassette at the transcriptional start site of *CG15556*, a 1.4 kb *Eco*RI fragment of pTL790 was subloned into pTL550 (pTL791). This subclone was outward PCR-amplified using primers tl_834F/tl_835R to generate a 4.5 kb amplicon, which was appended with a *Bst*EII site and was re-circularized at an *Aat*II site introduced through both primers (pTL792). The so modified 1.4 kb *Eco*RI fragment of pTL792 was re-introduced into pTL790 generating pTL793. An 1.7 kb fragment encoding the *LexA* cassette with primer-inserted *Aat*II and *Bst*EII sites was amplified off *pBSK-LexA-VP16-SV40* (Diegelmann *et al.* 2008) using primers tl_836F/tl_837R, cut with *Aat*II/*Bst*EII and inserted into pTL793 to generate the final *CG15556-LexA* reporter allele plasmid pTL794 (*attB^CC+^*, *FRT^+^*, *mW^+^*) (Table 2, Table 3).

**Table 2:** Primers used in this study

Primer	5′-3′ sequence
GSP#1	CACACGCTTACCAAGCACAA
GSP#2	TTCCACCGACACCAACAACA
tl_35R	TGCGACAGAGTGAGAGAGCAAT
tl_234F	CTCGCATATCTGGCTCTAAGACTTC
tl_631F	TTGAAGCTTACTAAATTGAAGCC
tl_634R	TCCAGAGTGCACTTTGCGGCAGA
tl_657F	CTTCGAGCACTGGCAAAAATTACG
tl_658R	AAACCGTAATTTTTGCCAGTGCTC
tl_659F	CTTCGAAGTGAGCGGGGAACTACT
tl_660R	AAACAGTAGTTCCCCGCTCACTTC
tl_661F	CTTCGATTGGCTGCCTGAAAGCGAG
tl_662R	AAACCTCGCTTTCAGGCAGCCAATC
tl_663F	CTTCGACTGACTCGAGTAGAGTTAT
tl_664R	AAACATAACTCTACTCGAGTCAGTC
tl_673F	ATCTCACCTGCAAGCTCGCGCTGTGTTCATCTACTCAAAGTAG
tl_674R	GAATCACCTGCAGAACTACAATTTTTGCCAGTGCTCTCCTCAC
tl_675F	GTACGCTCTTCCTATAGTTCCCCGCTCACTTCAAATTTA
tl_676R	TAGAGCTCTTCTGACGCATGAGACTAGACCCTGAACTTG
tl_678R	ATTGAATTAGATCCCGTACGATA
tl_679F	TGTGGTTTGTCCAAACTCATCA
tl_681F	ATCTCACCTGCAAGCTCGCACTTATACTTCTTATAGTCCAGTT
tl_682R	GAATCACCTGCAGAACTACGCTTTCAGGCAGCCAATAATCCAC
tl_683F	GTACGCTCTTCCTATACTCTACTCGAGTCAGTAGTAAAC
tl_684R	TAGAGCTCTTCTGACTCTGCAGCTTTGCGGTTCAGTGAC
tl_741F	GCGTTGGCAACGTCAGCGAGTCA
tl_742R	AATGCGGTATGTGAATGCGATAA
tl_743F	ATGTTGCCATAGAAATAAGTATT
tl_744R	TGACTAGTTGGTAGAAATTATGT
tl_768F	ATAGTTTAGCGGCCGCGAGTGGGCCTAGTGCTCGTATTTGA
tl_769R	AGGCGCGCCTATTGGCAACTGGCAAACTAAAATG
tl_814F	ATGGCTCGTACGGGATCTAATTCAATTAGAGACTAAT
tl_815R	GGAAGATCTTAAGATACATTGATGAGTTTGGACA
tl_822F	GGAAGATCTATCAAAATGAAGCTGCTGAGTAGTA
tl_823R	GCATGATACGCGCTAGCTCTAGAACTA
tl_824F	CTAGCTAGCATCGCCATGAATTTCAACTGGTGTG
tl_825R	GGAAGATCTGAATCAGGTGCCACAGCTGTTTGCA
tl_827F	ATAGTTTAGCGGCCGCACGTGGTTTTCGGTATATTCAGTTC
tl_828R	AGGCGCGCCACTTGGCTCGAAACGGTTAGAAGCT
tl_834F	GCTACCGACGTCTTATCTCGTAGGTCACCATCAAAATGCTGCTGTTTTTCTGGTGGATTGATGCTGCTGTTTTTCTGGTGGATTG
tl_835R	GACCGTGACGTCACTGCTATCTATTTTTAACTAATTT
tl_836F	GATGTCGACGTCATCAAAATGAAAGCGTTAACGGCCAGGCAAC
tl_837R	ATCGAAGGTGACCGATCCAGACATGATAAGATACATTG

**Table 3:** PCR primers for detection of attB/P recombination events

Primer	5′-3′ sequence
attL-CC.F	GGGCGTGCCCCCGAGTTCTCTC
attL-TT.F	GGGCGTGCCCTTGAGTTCTCTC
attP-CC.F	TGCCCCAACTGGGGTAACCTCC
attP-CC.R	ATAGGAACTTCCTACGCCCCCA
attP-TT.F	GTGCCCCAACTGGGGTAACCTTT
attP-TT.R	TACGAAGTTATCTACGCCCCCA
attP.R	CTACGCCCCCAACTGAGA
attR-TT.R	CGGGGAGCCCAAAGGTTACC
attR-CC.R	CGGGGAGCCCGGAGGTTACC

### Fly strains

#### Generated in this work:

LAT439, *w^1118^*; +; *attP^CC^ CG15556^KO^/TM3*, *Sb*; *(CG15556^KO^*, *w^-^)*
LAT483, *w^1118^*; +; *attP^CC^{CG15556-rescue mW^-^}CG15556^KO^/TM3*, *Sb*; *(CG15556^Rescue^*, *w^-^)*
LAT487, *w^1118^*; +; *attP^CC^{CG15556-LexA mW^-^}CG15556^KO^/TM3*, *Sb*; *(CG15556^LexA^*, *w^-^)*
LAT351, *w^1118^*; +; *attP^TT^ CG11318^KO^/TM3*, *Sb*; *(CG11318^KO^*, *DsRed^-^)*
LAT460, *w^1118^*; +; *attP^TT^{CG11318-rescue 3xP3-DsRed^-^}CG11318^KO^/TM3*, *Sb*; *(CG11318^Rescue^*, *DsRed^-^)*
LAT464, *w^1118^*; +; *attP^TT^{CG11318-p-GAL4 3xP3-DsRed^-^}CG11318^KO^/TM3*, *Sb*; *(CG11318^GAL4^*, *DsRed^-^)*
LAT443, *w^1118^*; +; *attP^CC^ CG15556^KO^*, *attP^TT^ CG11318^KO^/TM3*, *Sb*; *(CG15556^KO^ CG11318^KO^*, *w^-^ DsRed^-^)*
LAT540, w^1118^; +; attP^CC^{CG15556-rescue mW^-^}CG15556^KO^, attP^TT^{CG11318-rescue 3xP3-DsRed^-^}CG11318^KO^; (CG15556^Rescue^ CG11318^Rescue^, w^-^ DsRed^-^)
LAT584, w^1118^; +; attP^CC^{CG15556-LexA mW^-^}CG15556^KO^, attP^TT^{CG11318-p-GAL4 3xP3-DsRed^-^}CG11318^KO^ (CG15556^LexA^ CG11318^GAL4^, w^-^ DsRed^-^)

#### CRISPR/Cas9 targeting:

BDSC#56552, w^1118^; PBac{y^+mDint2^ = vas-Cas9}^VK00037^/CyO, P{w^+mC^ = Tb^1^}Cpr^CyO-A^;;
BDSC#55821, y^1^ M{vas-Cas9.RFP^-^}ZH-2A w^1118^;;;

(Both were gifts by Kate O’Connor-Giles and Jill Wildonger, University of Wisconsin, Madison.)

#### ΦC31 integration:

BL#32232, y^1^ w* P{y^+t7.7^ = nos-phiC31\int.NLS}X; P{y^+t7.7^ = CaryIP}su(Hw)^attP6^;;;
BL#40161, y^1^ M{vas-phiC31}ZH-2A w*;;;

#### Cre removal of loxP-flanked marker cassettes:

BDSC#851, y^1^ w^67c23^ P{y^+mDint2^ = Crey}1b;; D*/TM3, Sb^1^;

(Gift by Dan Hartl, Harvard University.)

#### FLP removal of FRT-flanked marker cassettes:

BDSC#6419, y^1^ w^1118^ P{ry^+t7.2^ = 70FLP}3F/Dp(1;Y)y^+^;; TM2/TM6C, Sb^1^;

(Gift by Michael Ashburner, University of Cambridge.)

#### Other strains:

GN86, y^1^ w*; wg^Sp-1^/CyO, P{Wee-P.ph0}Bacc^Wee-P20^; P{y^+t7.7^ w^+mC^ = 20xUAS-6xmCherry-HA}^attP2^;
GN83, y^1^ w*; PBac{y^+mDint2^ w^+mC^ = 20xUAS-6xGFP}^VK00018^/CyO, P{Wee-P.ph0}Bacc^Wee-P20^;; (Shearin *et al.* 2014)
RJK494, y^1^ w*; PBac{y^+mDint2^ w^+mC^ =13xLexAop2-6xmCherry-HA}^VK00018^/CyO, P{Wee-P.ph0}Bacc^Wee-P20^; Dr^1^/TM6C, Sb^1^ Tb^1^; (Shearin *et al.* 2014)
GN112, w*;;P{w^+mC^ = UAS-RFP.W}^3^, P{w[+m*]=lexAop-2xhrGFP.nls}3a
GN172, y^1^ w*; 20xUAS-6xGFP/CyO;lexAOp-myr-mCherry/TM6b;

### CRISPR/Cas9 gene targeting and ΦC31 assisted fly transgenesis

**Figure 2:**
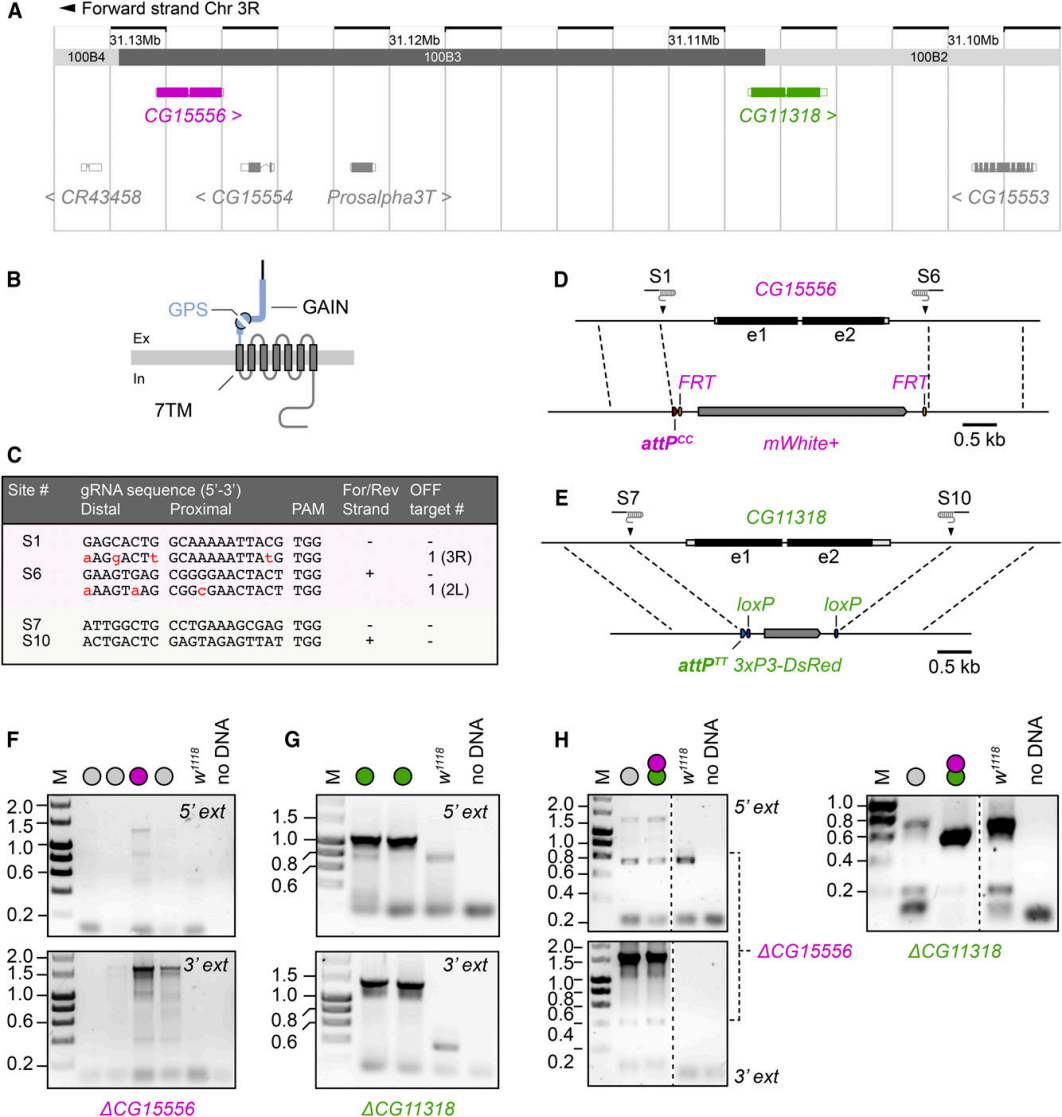
Gene targeting of linked loci *CG15556* and *CG11318*. (A) Genomic position of *CG15556* and *CG11318* on chromosome *III*. Note the presence of two intervening genes between the two aGPCR loci. (B) Molecular layout of the two aGPCRs encoded by *CG11318* and *CG15556*, which are marked by the heptahelical (7TM) and GPCR autoproteolysis-inducing (GAIN) domains, the latter of which contains the GPCR proteolysis site (GPS). Ex, extracellular, In, intracellular. (C) Target and potential off-target sequences (indicated with mismatches to the target sequence by lowercase red letters), location on forward/reverse DNA strand and number of potential off-targets (chromosome arm location of off-targets in brackets) for each gRNA used. (D,E) Targeting scheme for CRISPR/Cas9-mediated gene replacement for (D) *CG15556* and (E) *CG11318*. The upper line in each panel depicts the gene structure, while the lower line indicates the dimensions and elements of the replacement cassette with the selection marker (*mWhite*, *3xP3-DsRed*), *attP* site type (*attP^CC^* or *attP^TT^*) and recognition sites for the removal through site-specific Flippase or Cre recombinases (*FRT*, *loxP*). Dashed lines indicate homology arms of the HDR vector. Note the position of gRNAs that determine the positions of Cas9 cleavage above each gene locus and were used to replace *CG15556* (S1, S6) and *CG11318* (S7, S10). (F,G) PCR genotyping results on the genomic removal of (F) *CG15556* and (G) *CG11318*. Each lane represents an individual strain recovered after CRISPR/Cas9-targeting. 5′ and 3′ breakpoints in genomic DNA of strains with confirmed *CG15556* (magenta bullets; predicted PCR product sizes 5′=1,306 bp; 3′=1,628 bp) and *CG11318* (green bullets; predicted PCR product sizes 5′=1,033 bp; 3′=1,343 bp) removal were subsequently sequenced. (H) PCR genotyping results for separate detection of linked *CG15556* and *CG11318* removal in doubly targeted strains. Note: a different primer pair than employed in (F) was used to detect the presence of the *CG11318* deletion (predicted PCR product size = 658 bp). M, marker lane; gray bullets, unconfirmed strains; 5′/3′ ext = primer location outside the homology arms of the respective HDR vector; dashed lines indicate removal of intervening lanes from gel images.

All transgenesis steps were performed at Bestgene Inc. (Chino Hills, USA). For CRISPR/Cas9 mediated engineering of *CG11318* and *CG15556* single and double knockout/knockin alleles two gRNA and a matching HDR plasmid were injected into *w^1118^* flies carrying a germline-expressing Cas9 source (see Table 4) as described before (Gratz *et al.* 2014). Correct gene targeting was confirmed by subsequent sequencing of PCR fragments covering breakpoints between genomic/transgenic DNA amplified off genomic DNA of respective adult transgenic flies. To ensure no Cas9 cleavage occurred at the possible genomic OFF-targets for the *CG15556* targeting, suitably sized PCR fragments covering the OFFtargets were amplified from genomic DNA of *CG15556* single and double mutants and sequenced. The following primers were used for this: *CG15556* (5′: tl_743F/tl_35R; 3′: tl_234F/tl_744R), *CG11318* (5′: tl_741F/tl_678R; 3′: tl_679F/tl_742R; for the PCR shown in Figure 2C: tl_631F/tl_634R) (Table 2). Similarly, *attB^TT^* and *attB^CC^* carrying reporter plasmids were injected into flies with matching *attP^TT^* and/or *attP^CC^* landing pads, in which a germline expressing ΦC31 source was crossed, as indicated in Table 5. When selecting *3xP3-DsRed^+^* and *mW^+^* double recombinants it was noted that the *white* marker masks the presence of the *DsRed* fluorescence with exception of the ocelli. Ocellar *DsRed* expression, however, faithfully flags the presence of *DsRed^+^* transgenes in *w^+^* background and should be used to determine the presence of both markers (Figure 4).

**Table 4:** Details for CRISPR/Cas9 targeting of *CG15556* and *CG11318*

Target gene	gRNA	HDR repair with	Injection into	% Germline transmission	Transgenesis marker	% Marker removal
*CG15556*	S1, S6	*attP^TT^* (pTL649)	*vas-Cas9;;*; (#55821)	7 (4/55)	*loxP-DsRed-loxP*	N.A.
	S1, S6	*attP^CC^* (pTL724)	*vas-Cas9;;*; (#55821)	7 (4/60)	*FRT-mW-FRT*	100 (3/3)
	S1, S6	*attP^CC^* (pTL724)	*vas-Cas9;;CG11318^KO^;*	11 (6/55)	*FRT-mW-FRT*	100 (3/3)
*CG11318*	S7, S10	*attP^TT^* (pTL650)	; *vas-Cas9;*; (#56552)	1 (1/75)	*loxP-DsRed-loxP*	100 (5/5)

Indicated gRNAs were co-injected with respective HDR vectors (in brackets) encoding *attP^TT^* or *attP^CC^* landing pad sites into embryos containing germ-line expressing *vas-Cas9* transgenes (Bloomington Drosophila Genome Center stock numbers of source strains in brackets). Injected flies were crossed to *y^1^ w^1^* animals and progeny screened for germ-line transmission of w+ or DsRed+ eye markers (founder animals). At least one founder strain per targeting was sequenced to confirm the presence of the desired targeted lesion.

**Table 5:** Details for single and double ΦC31 mediated insertion of *CG15556* and *CG11318* transgenes using *attB^TT^* and *attB^CC^* recombination sites

Injection into	Injected with integration vector		% Germline transmission		Targeting precision *attB^XX^→attP^XX^*		Transgenesis marker	% Marker removal
	Encoding	*attB* site	Single	Double	Correct	Incorrect		
*CG15556^KO^ attP^CC^* (LAT439) *^1)^*	*CG15556^Rescue^* (pTL790)	*attB^CC+^*	4 (2/55)	N.A.	N.A.	N.A.	*FRT-mW*	100 (2/2)
*^1)^*	*CG15556p^LexA^* (pTL794)	*attB^CC+^*	5 (3/55)	N.A.	N.A.	N.A.	*FRT-mW*	100 (2/2)
*CG11318^KO^ attP^TT+^* (LAT351) *^1)^*	*CG11318^Rescue^* (pTL784)	*attB^TT+^*	9 (5/55)	N.A.	N.A.	N.A.	*loxP-DsRed*	100 (2/2)
*^1)^*	*CG11318p^GAL4^* (pTL789)	*attB^TT+^*	9 (5/55)	N.A.	N.A.	N.A.	*loxP-DsRed*	100 (2/2)
*CG15556^KO^ attP^CC+^*, *CG11318^KO^ attP^TT+^* (LAT443) *^1)^*	*CG15556^Rescue^*, − (pTL790 only)	*attB^CC+^*	4 (2/55)	N.A.	2	0	*FRT-mW*	100 (2/2)
*^1)^*	− , *CG11318^Rescue^* (pTL784 only)	*attB^TT+^*	5 (3/55)	N.A.	3	0	*loxP-DsRed*	100 (2/2)
*^2)^*	*CG15556^Rescue^* + *CG11318^Rescue^* (pTL790 + pTL784)	*attB^CC+^ attB^TT+^*	5 (3/55)	2 (1/55)* 4 (2/55)^#^	3	0	*FRT-mW + loxP-DsRed*	100 (2/2)
*^1)^*	*CG15556p^LexA^*, − (pTL794 only)	*attB^CC+^*	2 (1/65)	N.A.	1	0	*FRT-mW*	100 (1/1)
*^1)^*	− , *CG11318p^GAL4^* (pTL789 only)	*attB^TT+^*	9 (5/55)	N.A.	5	0	*loxP-DsRed*	100 (2/2)
*^2)^*	*CG15556p^LexA^* + *CG11318p^GAL4^* (pTL794 + pTL789)	*attB^CC+^ attB^TT+^*	9 (5/55)	2 (1/55)^#^	1	0	*FRT-mW + loxP-DsRed*	100 (2/2)

Single or double knockout flies were injected with individual or a combination of integration vectors containing either *attB^TT+^* or *attB^CC+^* recognition sites. The table lists efficiency and precision of ΦC31 mediated integration as well as the removal of transgenesis markers through Cre or FLP recombinases. The ΦC31 source crossed in for injections was either _1)_ *P{nos-phiC31\int.NLS}* or ^2)^ *M{vas-phiC31}ZH-2A*. *upon co-injection or #sequential injection of the plasmids.

### Imaging

Third instar larval whole-mount specimen were fixed in 4% paraformaldehyde in PBS for 30 min at room temperature, rinsed phosphate-buffered saline with 0.1% (v/v) Triton X100, mounted on a slide using Vectashield H1000 (Vector Laboratories, Burlingame, USA) and a coverslip, whose edges were sealed with nail varnish. Confocal images were obtained with a Leica SP8 system. Z-stacks were collected at 4 µm intervals while capturing the entire larvae in XY dimensions. Individual Z-planes and maximum projections of the Z-stacks were inspected for expression pattern analysis.

### Data availability

The fly strains described in this article are available upon request; the plasmids will be deposited at Addgene. Supplemental material available at Figshare: https://doi.org/10.25387/g3.6860723.

## Results

### Construction of an orthogonal attB^CC^/attP^CC^ pair

We have previously investigated structure-function relationships of the neuronal aGPCR homolog latrophilin/dCIRL using genomic engineering of the *dCirl* locus in *Drosophila* (Scholz *et al.* 2015; 2017). In quest of additional potential fly aGPCR homologs we identified two genes on chromosome *III* that contain open reading frames encoding seven transmembrane-spanning (7TM) and GPCR autoproteolysis-inducing (GAIN) domains, the combination thereof being the molecular tell-tale signature of the aGPCR family (Figure 2B) (Langenhan *et al.* 2013). *CG15556* and *CG11318* display high sequence conservation (data not shown) necessitating the construction of single and double knockout animals to account for possible functional redundancy. However, as both genes are closely linked on a 25 kb genomic fragment and separated through two additional genes (Figure 2A) we sought to remove each gene separately through CRISPR/Cas9-assisted homologous recombination.

To allow for later gene-specific rescue and modification of each locus independently through ΦC31-mediated integration, we generated a set of vectors encoding an *attB^CC^/attP^CC^* site pair that can function orthogonally to *attB^TT^/attP^TT^* sites contained in standard genomic engineering vectors (Huang *et al.* 2009; Gratz *et al.* 2013a; Gratz *et al.* 2014):

- A homology-directed repair vector harboring an *attP^CC^* site, in which the central crossover dinucleotide was changed from *TT* to *CC* (*pHD-attP^CC^-FRT-mW-FRT*). To facilitate the selection of recombinant flies that were also targeted with *pHD-DsRed-attP^TT^* (Gratz *et al.* 2014), the plasmid additionally contains a *FRT*-flanked *mini-White* selection cassette for removal through FLP recombinase expression rendering all main characteristics of the vector orthogonal to *pHD-DsRed-attP^TT^*. All other elements including the multiple cloning sites for homology arm insertion are identical to *pHD-DsRed-attP^TT^* (Figure S1A).
- An integration vector (*pGE-attB^CC^-FRT-mW*) containing an *attB^CC^* element, *mini-White* selection marker and *FRT* site (Figure S1B).

### CRISPR/Cas9 targeting of *CG11318* and *CG15556* with canonical attP^TT^ and novel attP^CC^ sites

*CG11318* and *CG15556* were individually targeted through CRISPR/Cas9-mediated homology directed repair (Gratz *et al.* 2014) (Figure 2C-E). Chimeric guide RNAs (gRNAs) for the gene targeting were selected to completely remove each gene, 5′ and 3′ UTRs and potential promoter regions (Figure 2C). Homology arms were about 1 kb in length for each HDR vector and placed to immediately edge the Cas9 cleavage sites (Figure 2D,E).

In a first round, *CG11318* and *CG15556* were individually targeted with a standard HDR plasmid (Gratz *et al.* 2014) replacing the genes with an *attP^TT^* docking site, whereas in a separate targeting round of *CG15556* an HDR vector encoding the *attP^CC^* variant was used. Cre-mediated *DsRed* cassette removal in *CG11318* recombinants and FLP-mediated *mini-White* excision (Golic and Lindquist 1989) from *CG15556*-targeted animals was performed yielding single *CG11318^KO^ attP^TT+^* and *CG15556^KO^ attP^CC+^* knockout/knockin founder animals for both aGPCR loci. Subsequent PCR genotyping and sequencing confirmed the correct insertion of the replacement cassettes into the *CG15556* (Figure 2F; Table 4) and *CG11318* (Figure 2G; Table 4) loci.

To generate a chromosome lacking both genes, we selected a *CG11318^KO^ attP^TT+^* founder strain, crossed it to a *vasa-Cas9* background and targeted *CG15556* by CRISPR/Cas9 as described above to yield *CG15556^KO^ attP^CC+^*, *CG11318^KO^ attP^TT+^* double mutant founders, which were verified by PCR genotyping and sequencing (Figure 2H; Table 4). *mW* and *DsRed* markers were subsequently removed from these recombinants through consecutive rounds of Cre and FLP recombination to yield *CG15556^KO^ attP^CC+^ mW*^-^, *CG11318^KO^ attP^TT+^ DsRed*^-^ founders (Table 4).

### Integration of attB^CC^ transgenes into attP^CC^ landing pads

We next tested whether the novel *attB^CC^/attP^CC^* pair can be used for ΦC31-mediated recombination. We selected a *CG15556^KO^ attP^CC+^* founder line and independently injected two constructs carrying cognate *attB^CC^* (*CG15556^Rescue^*; *CG15556p^LexA^*) for ΦC31 mediated integration and recovered 2 and 3 recombinant founder animals, respectively (Table 5). Sequencing of the genomic site of the *attB/attP* recombination for both transgenic integrants confirmed the formation of hybrid *attR^CC^* and *attL^CC^* sites on either side of the inserted DNA fragment (Figure 1C,D; Table S1). This demonstrates that the *attB^CC^/attP^CC^* pair with exchanged overlap dinucleotides allows for directional transgene incorporation in *Drosophila* (Figure 1B).

We continued to remove the *mini-White* marker cassette by standard FLP expression (Golic and Lindquist 1989) showing that the *pHDR-mW-attP^CC^-FRT* and its matching *pGE-attB^CC^-FRT-mW* partner vector can be used to complete ΦC31 assisted allele construction including transgenesis marker removal (Table 5).

### Orthogonality of attB^TT+^ and attB^CC+^ transgene integration

To evaluate the precision at which *attP^TT^* and *attP^CC^* sites, concomittantly present in the genome, are targeted we injected *CG15556^KO^ w^-^ attP^CC+^*, *CG11318^KO^ DsRed^-^ attP^TT+^* embryos expressing ΦC31 in the germline with either *CG15556p^LexA^ attB^CC^* or *CG11318p^GAL4^ attB^TT^* reporter vectors, and recovered independent integrants from each injection (Table 5).

After expanding a balanced stock from each of the resultant *CG15556p^LexA+^ mW^+^* or *CG11318p^GAL4+^ DsRed^+^* founder animals, genomic DNA was harvested and subjected to PCR genotyping and DNA sequencing to assess into which *attP* integration site the vector was inserted. We found that the accuracy of *attB^TT^→attP^TT^* and *attB^CC^→attP^CC^* integration was complete (5 correct integrants/5 recovered integrants for *CG11318p^GAL4^*; 1/1 for *CG15556p^LexA^*; Table 5). Notably, we observed no *attB^TT^→attP^CC^* or *attB^CC^→attP^TT^* recombinations in addition to correctly targeted integration events (0 wrong integrants/6 recovered integrants; Table 5).

**Figure 3:**
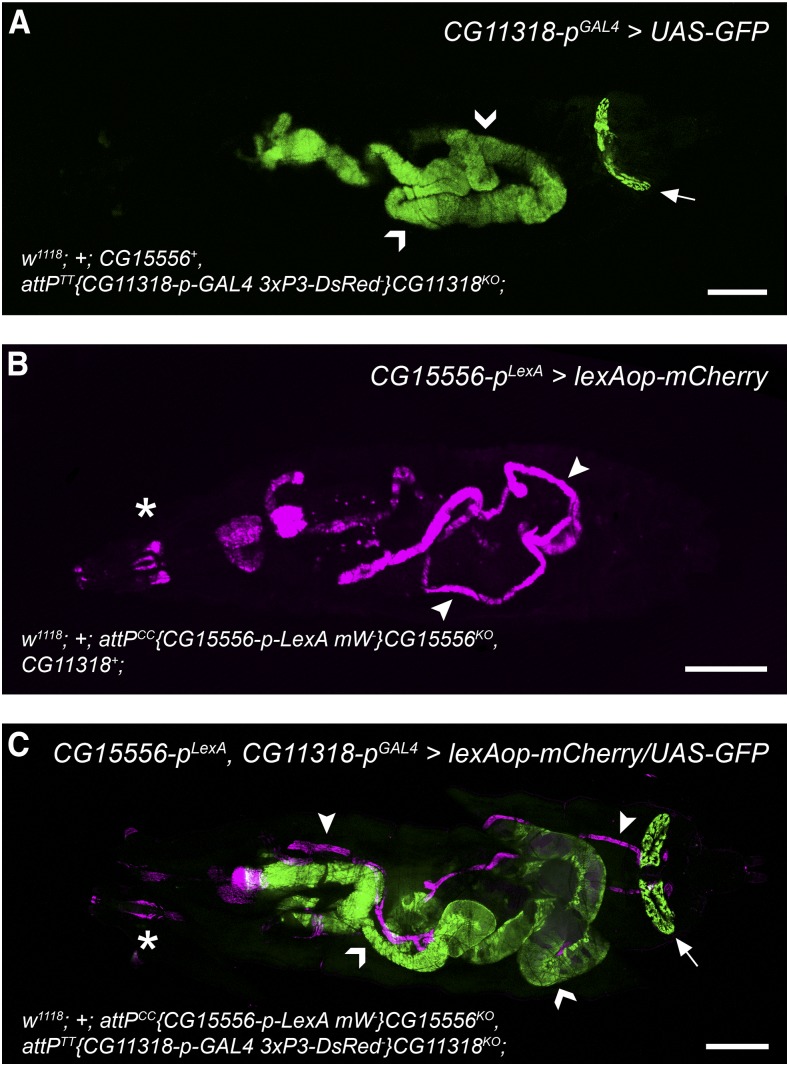
Transcriptional expression pattern of *CG15556* and *CG11318* in the third instar larva. The patterns were obtained through single (A) *CG11318-GAL4*, (B) *CG15556-LexA*, and (C) double *CG15556-LexA*, *CG11318-GAL4* reporter lines in third instar larvae. For clarity the genotype of the reporter carrying parent is given in the lower left corner of each panel. Anterior to the left, posterior to the right. Chevrons, gut; arrowheads, Malpighian tubules; arrow, anal pad; asterisk marks *CG15556* expression in neuronal or tracheal profiles in the head. Scale bars = 0.5 mm.

This was corroborated by expression analyses of *CG15556p^LexA^* or *CG11318p^GAL4^* reporter animals. Each *CG11318p^GAL4+^* founder stock was crossed to *20xUAS-6xmCherry-HA* or *20xUAS-6xGFP* partners and the expression pattern or their progeny was analyzed by fluorescence microscopical inspection. All offspring exhibited identical gene expression throughout the gastrointestinal canal at all developmental stages (5/5), and none displayed gene activity in the *CG15556* expression domain in the Malpighian tubule system (0/5; Figure 3A). Similar results were obtained for *CG15556p^LexA^* founder crosses with *13xLexAop2-6xmCherry-HA* reporters, which all showed expression in Malpighian tubules (1/1) but none in the gut (0/1; Figure 3B). Both receptor gene expression patterns confirm whole transcriptome microarray and RNAseq datasets made available through the Flyatlas (Chintapalli *et al.* 2007) and modENCODE (Graveley *et al.* 2011) projects.

In sum, this indicates that the overlap dinucleotide difference in both *attP* sites allows for a sufficiently high specificity of cognate *attB* vector integration using standard ΦC31 expression and injection protocols, and that both *attB*/*attP* pairs function orthogonally to each other. Nonetheless, future projects that will increase the sample size of parallel targetings with this approach are warranted to gain a definitive estimate on the specificity of both *attB*/*attP* site pairs.

### Integration of two transgenes in attP^TT+^ and attP^CC+^ animals

Based on these results we finally tested whether two transgenes, one endowed with an *attB^TT+^* and the other with an *attB^CC+^* site, can be genomically inserted in the same animal. To this end we repeated the ΦC31 recombinations in two regimes:

- Sequential *attP^TT^*/*attP^CC^* targeting: We selected single integrant strains of the transcriptional reporters *CG11318p^GAL4^* and *CG15556p^LexA^* that contained an unoccupied *attP^CC^* or *attP^TT^* site, respectively, and repeated the ΦC31 protocol with the other transgenic reporter not present yet. I.e. *CG15556^KO^ w^-^ FRT^+^ attP^CC^{CG15556p^LexA^ mW^+^ FRT^+^}*, *CG11318^KO^ DsRed^-^ loxP^+^attP^TT+^* animals were injected with a plasmid carrying CG11318p^GAL4^ attB^TT+^ DsRed^+^ loxP^+^, while CG15556^KO^ w^-^ FRT^+^ attP^CC+^, CG11318^KO^ DsRed^-^ loxP^+^ attP^TT^{CG11318p^GAL4^ DsRed^+^ loxP^+^} embryos received the CG15556p^LexA^ attB^CC+^mW^+^ FRT^+^ vector. Resulting recombinants were selected by the presence of both eye selection markers.
- Simultaneous *attP^TT^*/*attP^CC^* targeting: *CG15556^KO^ w^-^ FRT^+^ attP^CC+^*, *CG11318^KO^ DsRed^-^ loxP^+^attP^TT+^* founders were injected with both plasmids as in (*i*) in the same injection round.

We successfully recovered double recombinants with both regimes (Table 5) demonstrating that the dual targeting of two loci is feasible in succession but also simultaneously, although for the latter expectedly at the expense of efficiency (Table 5). The precision of *attB^TT+^* and *attB^CC+^* transgene integration into their respective genomic landing pads within the same genome was confirmed by PCR genotyping (Figure S2). In addition, we crossed a founder line carrying the linked *CG15556p^LexA^ CG11318p^GAL4^* transcriptional reporter alleles to a strain with matching *lexAop-mCherry* and *UAS-6xGFP* transgenes and observed expression domains for both genes in third instar larvae (Figure 3C) and adults (not shown) that were identical to the ones obtained from the single reporter expression assays (Figure 3A,B).

## Discussion

**Figure 4:**
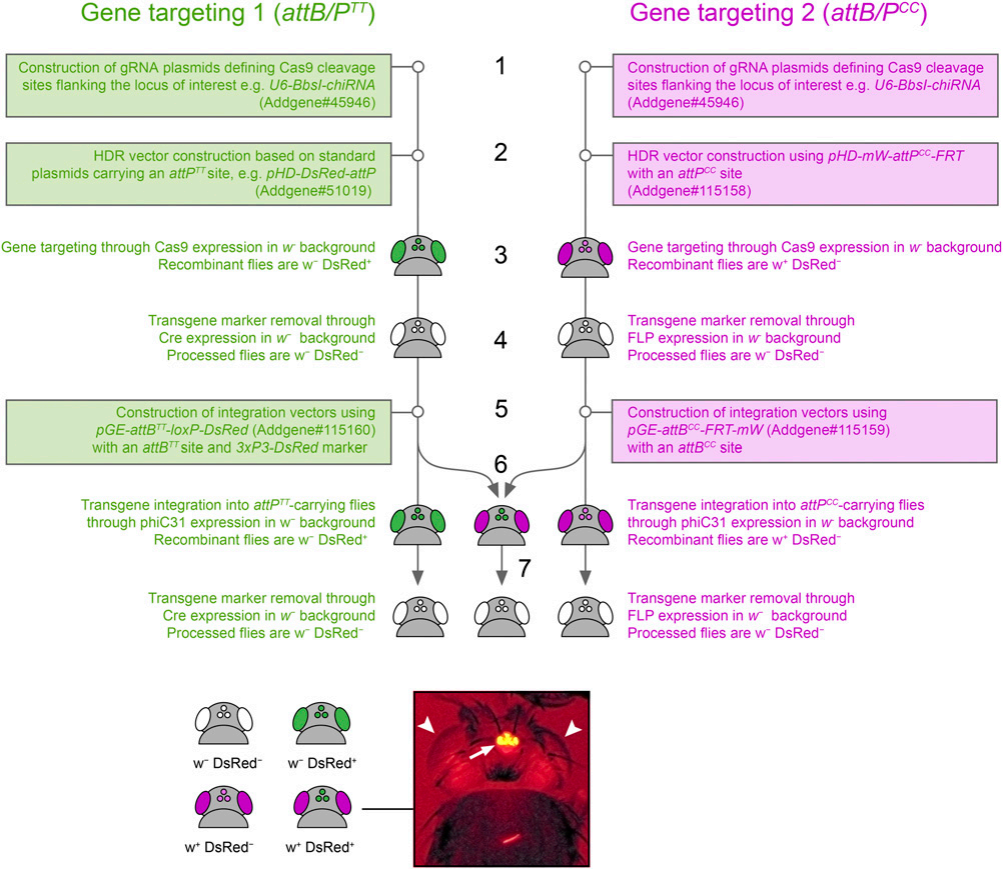
Work flow diagram highlighting vector use and eye color screening paradigm for identification of successful transgene integrations using two sets of orthogonal *attB/attP* sites. Steps 1-5 and 7 require the sequential targeting of the loci of interest to deposit both an *attP^TT^* and an *attP^CC^* site in the same genome. Once an *attP^TT^* + *attP^CC^* founder stock has been constructed, step 6 can be performed sequentially but, importantly, also in parallel for both targets allowing for high-throughput contemporaneous engineering of two genes. Steps that entail plasmid construction are boxed and suitable vector backbones are indicated. Note that in animals that have co-received transgenes marked with *mW^+^* and *3xP3-DsRed^+^* the latter marker is masked by the w^+^ color in the eye (see arrowheads in fluorescence micrograph). However, the strong ocellar DsRed expression reliably indicates the presence of *3xP3-DsRed^+^* transgenes (arrow in micrograph). Genotype of the photographed fly was *w^1118^*; *+*; *attP^CC^{CG15556-rescue mW^+^}CG15556^KO^*, *attP^TT^{CG11318-rescue 3xP3-DsRed^+^}CG11318^KO^*.

Here, we demonstrate a simple, easily adaptable and efficient system for the separate and repeated manipulation of two linked genetic loci in *Drosophila* (Figure 4). This protocol capitalizes on the currently most widely used genomic engineering toolkit for this model species, the ΦC31 integrase assisted transgenesis method (Bischof *et al.* 2007). Altogether, the results indicate that the specificity, directionality and recombination efficiency of the orthogonal *attB^CC^/attP^CC^* site pair introduced here can be used in conjunction with the canonical *attB^TT^/attP^TT^* system and established ΦC31 resources to handle two transgenesis targets independently and simultaneously.

This confirms results obtained in *E. coli* using an array of similar orthogonal *attB/attP* site pairs including the *attB^CC^/attP^CC^* version used here in *Drosophila* (Colloms *et al.* 2014). The successful recombination of *attB^CC^/attP^CC^* elements suggests that also in metazoan cells the parallel alignment of the recombination sites during synapsis of the DNA strands is not influenced by the nature of the crossover nucleotides as long as they are reverse-complementary to each other (Smith *et al.* 2004b). Likewise, our work further implies that – in addition to *attB/attP* sites carrying *TT/AA* and *CC/GG* sequences – other *attB/attP* pairs with asymmetric central overlap dinucleotides (*GT/CA*; *CT/GA*; *TC/AG*; *CA/GT*) will likely function as precise and independent, *i.e.*, orthogonal, targeting addresses for ΦC31 integrase as established in *E. coli* (Smith *et al.* 2004b; Colloms *et al.* 2014). Therefore, this rationale can expand the parallel modifiability of genes in *Drosophila* to up to six genomic locations by a simple modification to the *attP* landing pads and associated *attB*-containing integration vectors.

Notably, site-directed transgenesis through integrases orthogonal to ΦC31 and with specific recognition sequences offers an alternative approach for the separate handling of multiple loci during genomic engineering. Several other such recombinases were shown to operate in *Drosophila* (reviewed in (Venken *et al.* 2016)). For example, recently, the mycobacteriophage integrase system BxbI was introduced for *Drosophila* transgenesis and shown to operate in parallel to ΦC31 (Huang *et al.* 2011; Voutev and Mann 2017). However, the combination of two integrases requires successive rather than parallel integration of transgenes doubling the processing time required for the establishment of doubly recombinant fly strains. In addition, the system is not widely used yet by academic laboratories and not offered by commercial suppliers for fly transgenesis services limiting the potential of this elegant tool and the principle of establishing orthogonality to ΦC31 transgenesis at the level of the employed integrase systems. Nonetheless, the insights and feasibility of alternative *attB/attP* site usage by ΦC31 provided in the current work is molecularly separate from those tools. It may thus even be combined with non-ΦC31 integrase mediated transgenesis tools, and in addition also be adopted by non-ΦC31 systems to expand their own recombinatorial transgene→target logic.
